# Identification and characterization of linear B-cell epitopes on African swine fever virus H171R protein

**DOI:** 10.1128/spectrum.01411-24

**Published:** 2024-10-23

**Authors:** Cheng Xin, Zhuting Chen, Jingming Zhou, Yumei Chen, Yankai Liu, Hongliang Liu, Chao Liang, Xifang Zhu, Yanhua Qi, Gaiping Zhang, Aiping Wang

**Affiliations:** 1 School of Life Sciences, Zhengzhou University, Henan, Zhengzhou, China; 2 Longhu Laboratory, Henan, Zhengzhou, China; 3 Henan Key Laboratory of Immunobiology, Henan, Zhengzhou, China; 4 School of Advanced Agricultural Sciences, Peking University, Beijing, China; 5 College of Veterinary Medicine, Henan Agricultural University, Henan, Zhengzhou, China; National Chung Hsing University, Taiwan, China

**Keywords:** African swine fever virus, H171R protein, monoclonal antibody, B-cell epitope

## Abstract

African swine fever virus (ASFV) is a highly contagious and often fatal pathogen that poses a significant threat to the swine industry worldwide. The H171R protein, a structural component of ASFV, plays crucial roles in viral assembly, host cell entry, and modulation of the host immune response. This study aimed to comprehensively characterize the linear B-cell epitopes on the H171R protein to facilitate the development of diagnostic tools and subunit vaccines against ASFV. A combined approach involving bioinformatics analysis and experimental techniques was employed. The recombinant H171R protein was expressed and purified, and specific monoclonal antibodies were generated through immunization and hybridoma technology. Systematic epitope mapping using overlapping peptide fragments and alanine-scanning mutagenesis revealed four minimal linear epitopes: ^84^HPLLPYQQSSDEQP^97^, ^93^SDEQPMMPYQQPPG^106^, ^111^PYEQIYHKKHASQQ^124^, and ^129^LNDYYQHILALGDED^143^. The identified epitopes exhibited strong immunogenicity, as demonstrated by their reactivity with ASFV-positive swine sera. Critical amino acid residues within each epitope were identified through mutational analysis. Structural modeling and visualization of the H171R protein provided insights into the spatial distribution and accessibility of the epitope regions. These findings contribute to a better understanding of the H171R protein’s antigenic properties and lay the foundation for developing effective diagnostic assays and subunit vaccines against African swine fever.

**IMPORTANCE:**

African swine fever virus (ASFV) poses a severe threat to the global swine industry. This study characterizes linear B-cell epitopes on the crucial ASFV H171R protein, facilitating the development of improved diagnostics and subunit vaccines. Four immunogenic epitopes were identified, offering valuable information for designing sensitive diagnostic assays and potential subunit vaccine candidates. By advancing the understanding of H171R’s antigenic landscape, this research contributes to controlling ASFV’s devastating impacts, safeguarding the swine industry, and ensuring food security.

## INTRODUCTION

African swine fever (ASF) is a highly contagious and often fatal viral disease affecting domestic and wild pigs worldwide, causing substantial economic losses to the swine industry (1). The causative agent, African swine fever virus (ASFV), is a large, double-stranded DNA virus that encodes more than 150 proteins involved in various aspects of viral replication and pathogenesis (2). Currently, there are no effective vaccines or treatments available for ASF, and disease control relies solely on strict biosecurity measures and culling of infected herds (3).

Among the ASFV structural proteins, H171R has garnered significant interest due to its multifunctional roles in virus assembly, host cell entry, and modulation of the host immune response (4, 5). As a surface-exposed component of the viral particle, the H171R protein represents a promising target for developing diagnostic assays and subunit vaccines against ASFV (6). However, a comprehensive understanding of its immunogenic epitopes is crucial for exploiting its potential applications (7).

B-cell epitopes are specific structural regions on an antigen that are recognized and bound by antibodies. They can be classified into linear (continuous) and conformational (discontinuous) epitopes (8, 9). Mapping B-cell epitopes is a fundamental step in vaccine design and antibody engineering (10).

In this study, we employed a combination of bioinformatics analysis and experimental approaches to comprehensively identify and characterize the linear B-cell epitopes on the ASFV H171R protein. Through the expression and purification of the recombinant H171R protein, generation of specific monoclonal antibodies (mAbs), and systematic epitope mapping using overlapping peptide fragments, we aimed to delineate the minimal linear epitope sequences and their critical residues. The immunodominance of the identified epitopes was further validated using ASFV-positive swine sera. Additionally, a structural homology model of H171R was constructed to provide insights into the spatial distribution and potential functional implications of the identified epitopes. Our findings lay the foundation for future studies on the biological functions of the H171R protein and facilitate the development of diagnostic tools and subunit vaccines against ASF.

## MATERIALS AND METHODS

### Bacterial strains, plasmids, and cell lines

*Escherichia coli* DH5α (Invitrogen, Carlsbad, CA, USA) was employed for plasmid amplification and cloning procedures. The prokaryotic expression vector pET-32a(+) (Novagen, Madison, WI, USA) was utilized for the expression of recombinant H171R protein. BL21(DE3) competent cells (Invitrogen) were used for the production of the recombinant protein. SP2/0 myeloma cells (American Type Culture Collection, Manassas, VA, USA) were maintained in RPMI-1640 Medium (Gibco, Grand Island, NY, USA) supplemented with 10% fetal bovine serum (Gibco), 100 U/mL penicillin, and 100 µg/mL streptomycin (Gibco). The cells were incubated at 37°C in a humidified atmosphere containing 5% CO_2_. These SP2/0 myeloma cells were used as fusion partners for the generation of hybridomas secreting monoclonal antibodies.

### Reagents

Restriction endonucleases *BamH*I and *Xho*I, T4 DNA ligase, DNA polymerase, deoxy-ribonucleoside triphosphate (dNTP), prestained protein molecular weight markers, and Precision Plus Protein Unstained Standards were procured from Thermo Fisher Scientific (USA). Urea, Tris, and SDS were obtained from Sigma-Aldrich (USA). The low-molecular-weight range (10–150 kDa) prestained protein ladder was acquired from Bio-Rad (USA). The anti-His tag monoclonal antibody was purchased from Abcam (UK), while horseradish peroxidase (HRP)-conjugated goat anti-mouse IgG was obtained from Sigma-Aldrich. ASFV-positive swine sera were provided by the China Institute of Veterinary Drug Control.

### Cloning, expression, and purification of the recombinant H171R protein

The full-length H171R gene was amplified by PCR, and the purified product was digested with *BamH*I and *Xho*I before ligation into the pET-32a(+) vector. The recombinant plasmid pET-32a(+)-H171R was transformed into *E. coli* BL21(DE3) cells. Positive clones were induced for recombinant protein expression in Luria-Bertani (LB) media with 0.1–1.0 mM isopropyl β-D-Thiogalactoside (IPTG) at 16°C and 37°C for 15 h. SDS-PAGE analysis determined the optimal induction conditions for recombinant H171R protein expression.

### Western blot analysis of H171R immunogenicity

To assess the immunogenicity of recombinant H171R, western blot (WB) analysis was conducted. The purified protein was separated by SDS-PAGE, transferred onto a polyvinylidene difluoride membrane, and blocked with 5% skim milk in Tris-buffered saline containing 0.1% Tween-20 for 1 h. The membrane was incubated with ASFV-positive swine sera (1:1,000) overnight at 4°C, followed by HRP-conjugated goat anti-swine IgG (1:5,000) for 1 h. Immunoreactive bands were visualized using an enhanced chemiluminescence detection system (11).

### Mouse immunization procedure and cell fusion

Balb/C mice were immunized with 50 µg of purified H171R protein emulsified with Freund’s complete adjuvant, followed by three booster immunizations at 2-week intervals, each with 30 µg of antigen. One week after the final booster, the antibody titer in mouse antisera was determined by an enzyme-linked immunosorbent assay (ELISA) (12). The mouse with the highest antibody titer was selected and injected intraperitoneally with H171R protein in phosphate-buffered saline (PBS). After 3 days, the spleen was removed, ground to obtain a single-cell suspension, and mixed with SP2/0 myeloma cells at an 8:1 ratio. The cell mixture was centrifuged, and the pellet was treated with 50% PEG 1500 solution. The cells were then resuspended in a serum-free complete medium containing hypoxanthine–aminopterin–thymidine, dispensed into 96-well plates, and incubated at 37°C with 5% CO_2_ for 10 days. Culture supernatants from single clones were screened by ELISA, and positive clones were expanded by subculturing.

### Monoclonal antibody production and characterization

Positive hybridoma clones were expanded and injected into pristane-primed Balb/C mice for ascites production. The mAbs were purified from ascitic fluid using caprylic acid–ammonium sulfate precipitation (13). Purified mAbs were characterized by SDS-PAGE and western blot for purity and specificity. Titers were determined by ELISA, isotypes by a mouse monoclonal antibody isotyping kit, and affinity constants (Ka) by ELISA using Beatty et al.’s method (14). The mAbs’ reactivity against recombinant H171R protein was evaluated by indirect ELISA. Microtiter plates were coated with H171R protein (1 µg/mL) overnight at 4°C, blocked with 5% skim milk in phosphate buffered saline with tween-20 (v/v, 0.05%) (PBST), and then incubated with serial mAb dilutions for 1 h at 37°C. After washing, plates were incubated with HRP-conjugated goat anti-mouse IgG (1:5,000) for 1 h at 37°C. The reaction was developed using a 3, 3′, 5, 5′-tetramethylbenzidine (TMB) substrate, and absorbance was measured at 450 nm.

### Expression and characterization of linear H171R protein fragments

Multiple overlapping peptide fragments along the H171R protein amino acid sequence were designed using DNAStar software. The fragments were digested and cloned into the pET-32a(+) vector for expression and purification in *Escherichia coli* BL21(DE3). Dot-ELISA and indirect ELISA methods were employed to assess the reactivity of the purified linear peptide fragments with the prepared monoclonal antibodies, determining the recognition region for each monoclonal antibody. Based on the initial results, shorter peptide fragments were redesigned to narrow down the recognition region, and the above steps were repeated until the minimal linear epitope region was obtained.

### Alanine scanning mutagenesis analysis

To validate critical amino acid residues’ roles, alanine scanning mutagenesis was used. Overlapping extension PCR substituted each residue’s codon with alanine. Alanine residues were replaced with glycine, as glycine is more structurally similar to alanine and less likely to disrupt the protein structure (15, 16). This generated single-point mutant epitope peptides, which were cloned into pET-32a(+) vector, expressed, and purified in BL21(DE3). Indirect ELISA and western blotting analyzed each mutant peptide’s reactivity with monoclonal antibodies, evaluating critical residues’ importance. A significant decrease in antibody binding after mutation indicated the residue’s crucial role in maintaining the epitope structure and antigenicity.

### Immunogenicity assessment of the identified epitopes

To evaluate the immunogenicity of the identified epitopes, ELISA and western blotting were employed to analyze the reactivity of the different epitope peptides with ASFV-positive pig sera.

### Spatial localization and visualization of the epitope regions

The online tool AlphaFold2 was utilized to perform homology modeling of the three-dimensional structure of the H171R protein based on sequence homology (17). The identified epitopes and critical amino acid residues were mapped onto the structural model, and the PyMOL molecular visualization software was used to present the distribution of the epitopes on the three-dimensional protein structure, providing insights into the spatial relationships and accessibility of the epitope regions.

### Statistical analysis

The data obtained from the experiments were analyzed using appropriate statistical methods. All experiments were performed in triplicate, and the results were expressed as the mean ± standard deviation. Statistical significance was determined by one-way analysis of variance followed by Tukey’s *post hoc* test for multiple comparisons. Differences were considered statistically significant at *P* < 0.05. GraphPad Prism software (version 9.0, GraphPad Software Inc., San Diego, CA, USA) was utilized for statistical analyses and data visualization.

## RESULTS

### Expression, purification, and immunogenicity of the H171R recombinant protein

**Fig 1:**
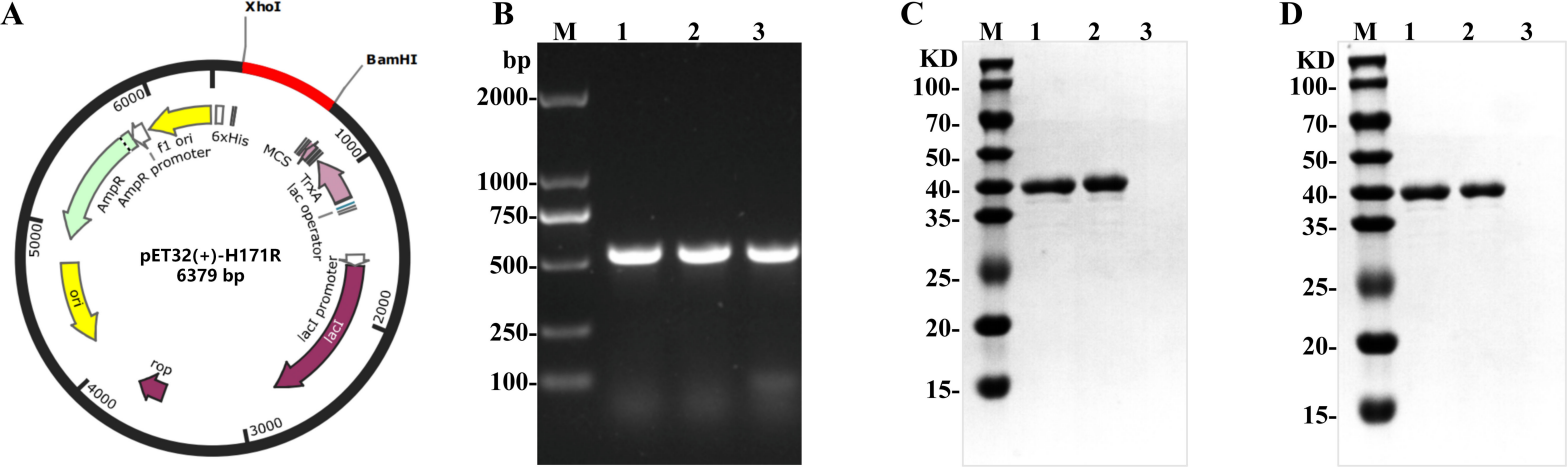
Cloning, expression, purification, and immunoreactivity of the H171R recombinant protein. (**A**) Schematic representation of the H171R gene cloning into the pET-32a(+) vector. (**B**) Agarose gel electrophoresis showing the amplification of the H171R gene. (**C**) Western blot analysis of the purified H171R recombinant protein using a mouse anti-His tag primary antibody and an HRP-conjugated goat anti-mouse secondary antibody. (**D**) Western blot demonstrating the specific binding of the purified H171R recombinant protein to ASFV-positive pig sera.

As shown in Fig. 1A and B, following double digestion, the H171R encoding gene fragment was cloned into the pET-32a(+) vector. Positive recombinant clones were verified by sequencing to confirm the correct insertion of the gene fragment. Large-scale expression of the H171R recombinant protein was performed under optimal conditions, and the soluble protein was purified using nickel-affinity chromatography. Western blotting results showed that the eluted fractions were primarily enriched with a protein band of approximately 40 kDa (anti-His monoclonal antibody), consistent with the theoretical molecular weight of the H171R protein (37.7 kDa) (Fig. 1C). The concentration of the purified H171R recombinant protein, as determined by the Bradford assay, reached 1.8 mg/mL. Western blotting results demonstrated that the purified H171R recombinant protein could specifically bind to a commercially available ASFV-positive pig sera (Fig. 1D), indicating its good immunoreactive activity and suitability as an immunogen for inducing the production of corresponding antibodies.

### Mouse serum antibody titer determination and hybridoma screening

**Fig 2:**
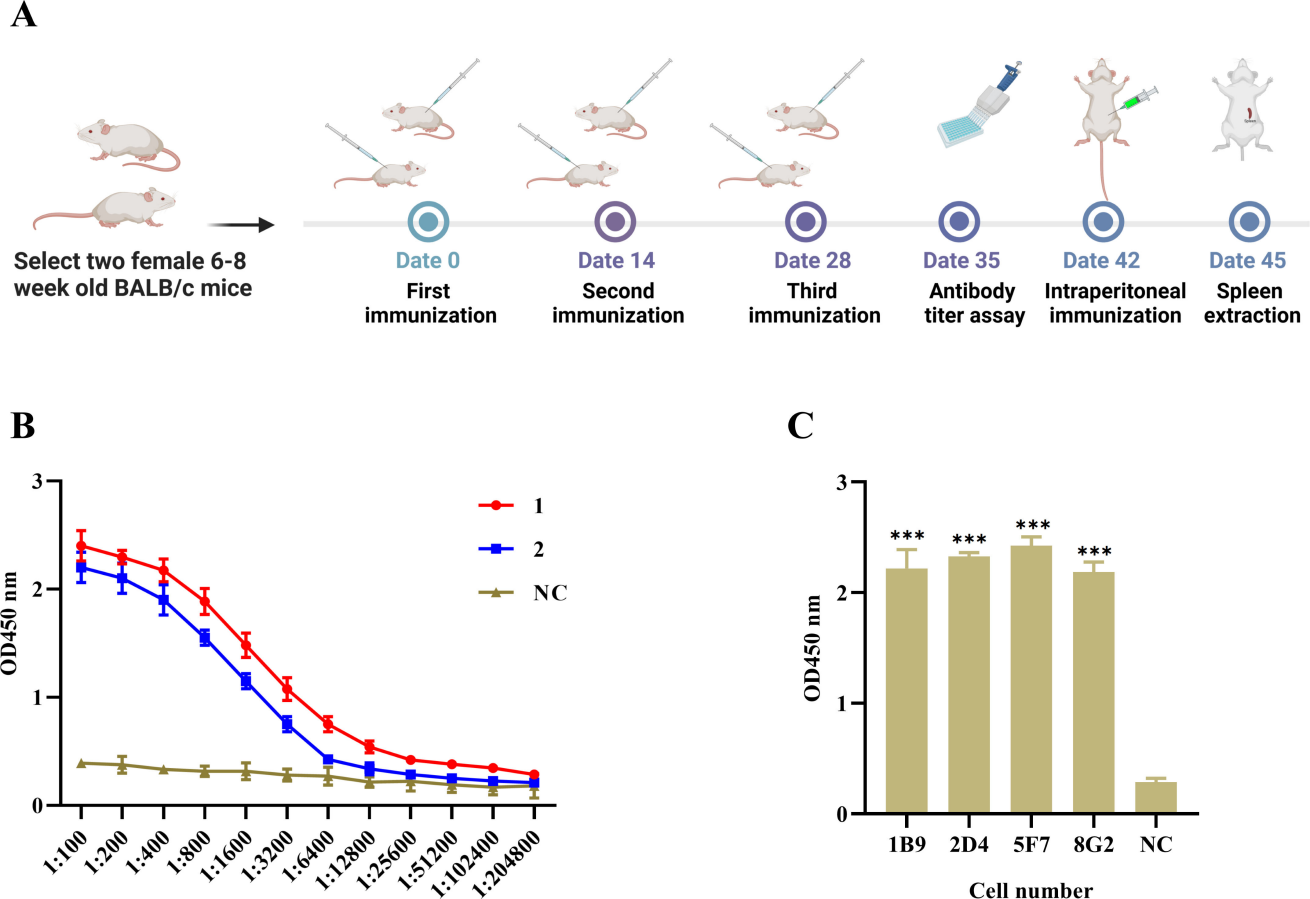
Mouse immunization, serum antibody titer determination, and hybridoma screening. (**A**) Immunization schedule for Balb/C mice using the purified H171R protein emulsified with Freund’s adjuvant. (**B**) Indirect ELISA results showing the antibody titers in mouse antisera 1 week after the third immunization. (**C**) ELISA screening of positive hybridoma cell lines against the H171R protein after limiting dilution and rescreening.

As shown in Fig. 2A, purified H171R protein was emulsified with Freund’s adjuvant and used to immunize 6–8-week-old Balb/C mice. Indirect ELISA was employed to determine the antibody titers in mouse antisera. Results indicated that some mice developed high titers, with two mice (designated as #1 and #2) exhibiting titers of 1:1.024 × 10^5^ and 1:5.12 × 10^4^, respectively, 1 week after the third immunization (Fig. 2B). Single-cell suspensions were prepared from the spleens of high-titer mice and fused with SP2/0 myeloma cells. Two weeks later, initial ELISA screening identified 12 positive hybridoma cell lines against the H171R protein. After limiting dilution and rescreening, four stable positive clones were obtained and named 1B9, 2D4, 5F7, and 8G2 (Fig. 2C).

### Preparation and identification of monoclonal antibodies

**Fig 3:**
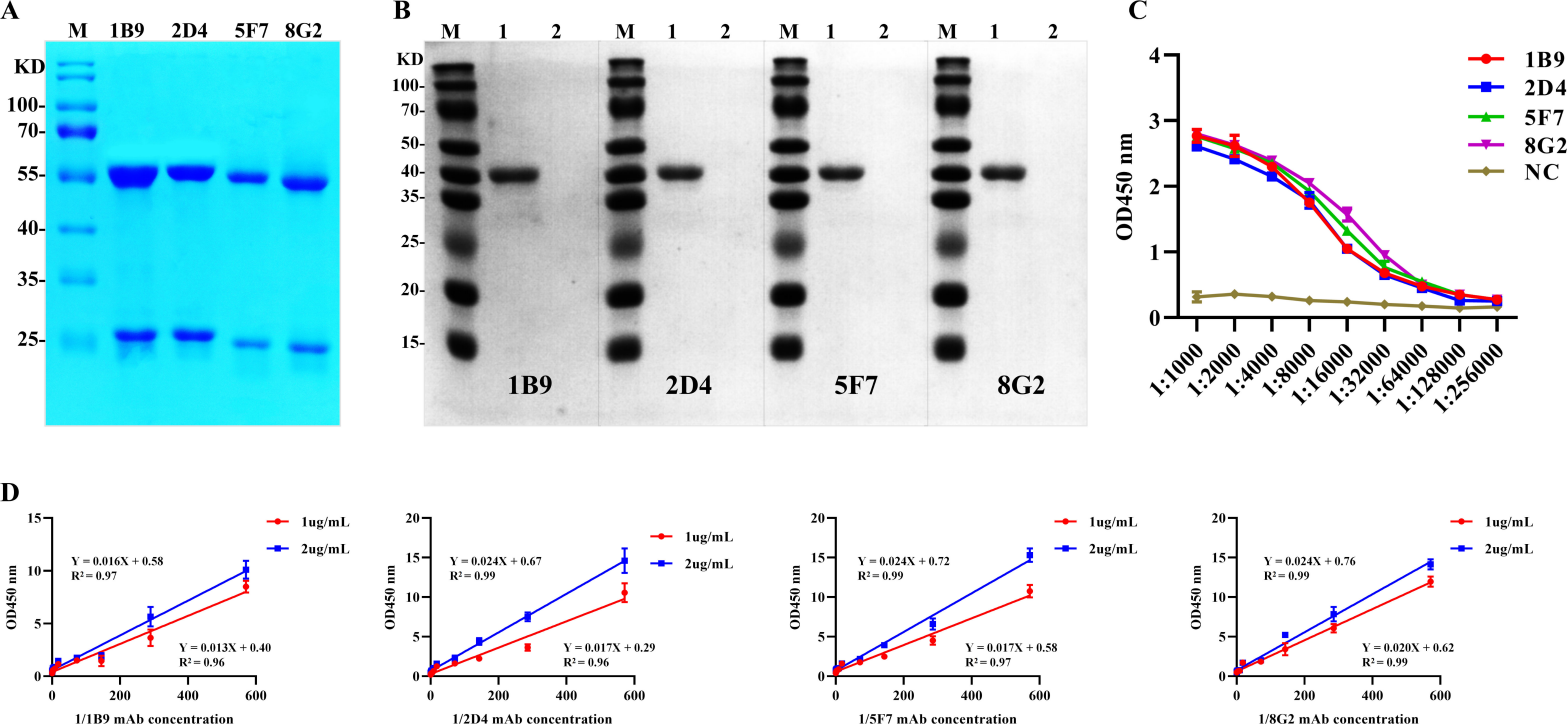
Preparation and identification of monoclonal antibodies. (**A**) SDS-PAGE analysis of the purified monoclonal antibodies showing heavy and light chain bands. (**B**) Western blot confirming the specific recognition of the H171R recombinant protein by the monoclonal antibodies. Lane M represents the protein marker, lane 1 contains the H171R protein, and lane 2 contains the TrxA tag protein as a negative control. (**C**) Indirect ELISA results demonstrating the titers of the four monoclonal antibodies (1B9, 2D4, 5F7, and 8G2). NC represents the negative control mouse serum. (**D**) Affinity determination of the monoclonal antibodies using the formula Kaff = (*n* − 1)/2(*n*[Ab′]*t* − [Ab]*t*).

The SDS-PAGE results of the antibodies purified by the acetic acid–ammonium sulfate method (Fig. 3A) showed a heavy chain band and a light chain band at approximately 55 and 25 kDa, respectively. The purity of the antibody bands, as determined by gel system lane analysis, reached 95%. Western blotting results confirmed that the monoclonal antibodies from these four positive clones could recognize the H171R recombinant protein, while showing no specific binding to a TrxA-tagged protein (Fig. 3B). The titers of the four monoclonal antibodies were determined by indirect ELISA. The results showed that the titers of 1B9, 2D4, 5F7, and 8G2 were 1:2.56 × 10^5^, 1:1.28 × 10^5^, 1:1.28 × 10^5^, and 1:1.28 × 10^5^, respectively (Fig. 3C). According to the formula Kaff = (*n* − 1)/2(*n*[Ab′]*t* − [Ab]*t*), the affinities of the 1B9, 2D4, 5F7, and 8G2 antibodies were calculated to be 4.75 × 10^9^ L/mol, 1.67 × 10^10^ L/mol, 3.84 × 10^9^ L/mol, and 2.91 × 10^9^ L/mol, respectively (Fig. 3D; Table 1).

**TABLE 1:** Characteristics of mAbs

MAbs	Epitope	mAb subtypes	Titers	Affinity constant “Ka” (L/mol)
1B9	Linear	IgG1, Kappa	1:2.56 × 10^5^	4.75 × 10^9^ L/mol
2D4	Linear	IgG2a, Kappa	1:1.28 × 10^5^	1.67 × 10^10^ L/mol
5F7	Linear	IgG1, Kappa	1:1.28 × 10^5^	3.84 × 10^9^ L/mol
8G2	Linear	IgG2a, Kappa	1:1.28 × 10^5^	2.91 × 10^9^ L/mol

### Truncated expression and identification of H171R protein

**Fig 4:**
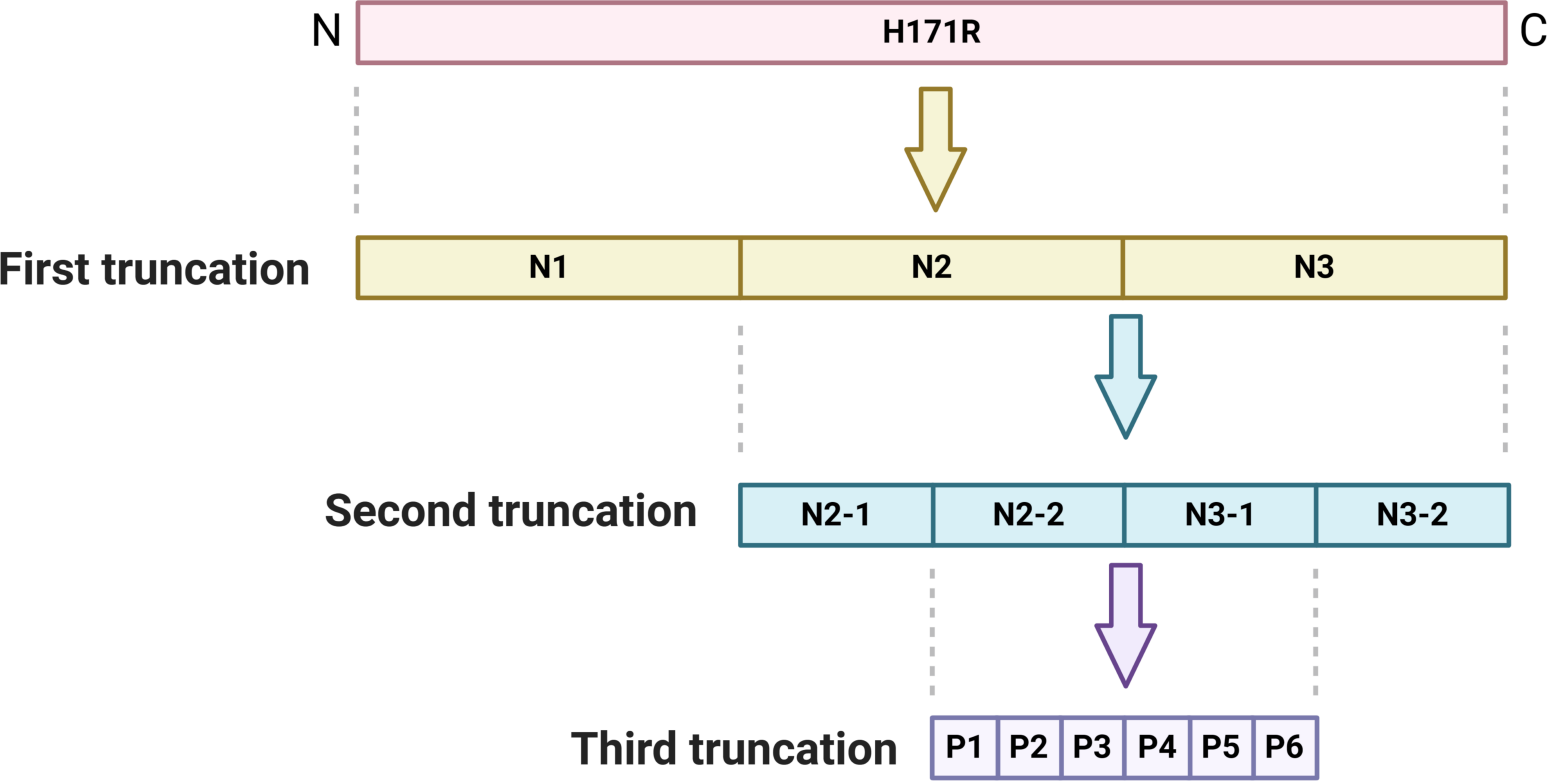
Schematic representation of the sequential truncation of the H171R protein into smaller fragments for epitope mapping.

**Fig 5:**
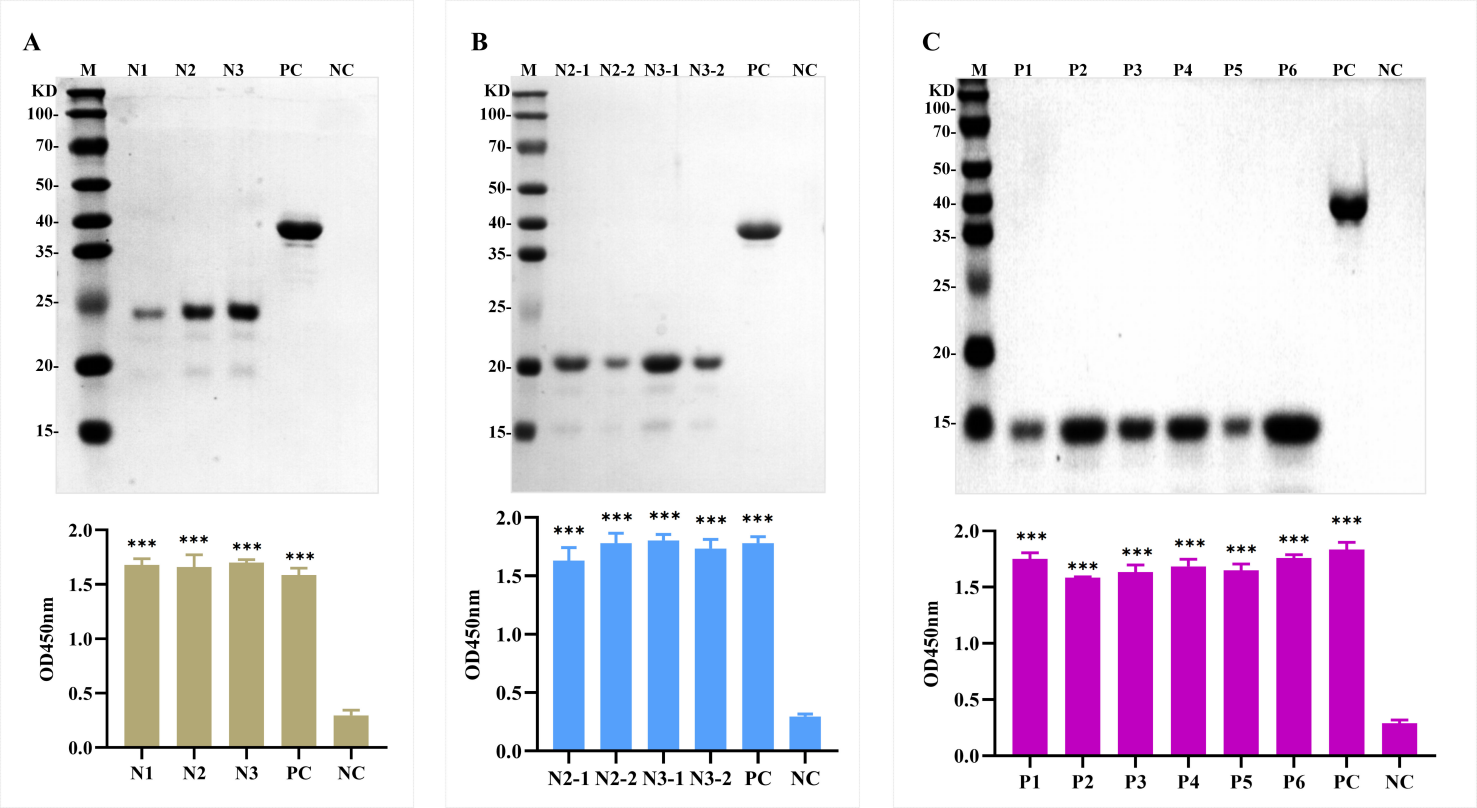
Truncated expression and identification of H171R protein fragments. (**A**) WB and indirect ELISA results demonstrating the successful expression of N1, N2, and N3 proteins in the first stage of truncation. (**B**) WB and indirect ELISA results verifying the successful expression of N2-1, N2-2, N3-1, and N3-2 proteins in the second stage of truncation. (**C**) WB and indirect ELISA results confirming the successful expression of P1, P2, P3, P4, P5, and P6 proteins in the third stage of truncation. In all panels, PC represents the full-length H171R protein as a positive control, and NC represents BSA protein without a His tag as a negative control.

As illustrated in Fig. 4, H171R underwent three stages of sequential truncation. In the first stage, the full-length H171R protein was truncated into three segments (N1–N3). WB and indirect ELISA (with mouse anti-His antibody as the primary antibody and HRP-labeled goat anti-mouse as the secondary antibody) collectively demonstrated the successful expression of N1, N2, and N3 proteins (Fig. 5A). In the second stage, the N2 and N3 regions, which reacted with the antibody, were further truncated into four segments (N2-1, N2-2, N3-1, and N3-2). WB and indirect ELISA (with mouse anti-His antibody as the primary antibody and HRP-labeled goat anti-mouse as the secondary antibody) jointly verified the successful expression of N2-1, N2-2, N3-1, and N3-2 proteins (Fig. 5B). In the third stage, the N2-2 and N3-1 regions, which exhibited reactivity with the antibody, were further truncated into six segments (P1, P2, P3, P4, P5, and P6). WB and indirect ELISA (with mouse anti-HIS antibody as the primary antibody and HRP-labeled goat anti-mouse as the secondary antibody) collectively confirmed the successful expression of P1, P2, P3, P4, P5, and P6 proteins (Fig. 5C).

### Screening of linear B-cell epitope regions on the H171R protein

**Fig 6:**
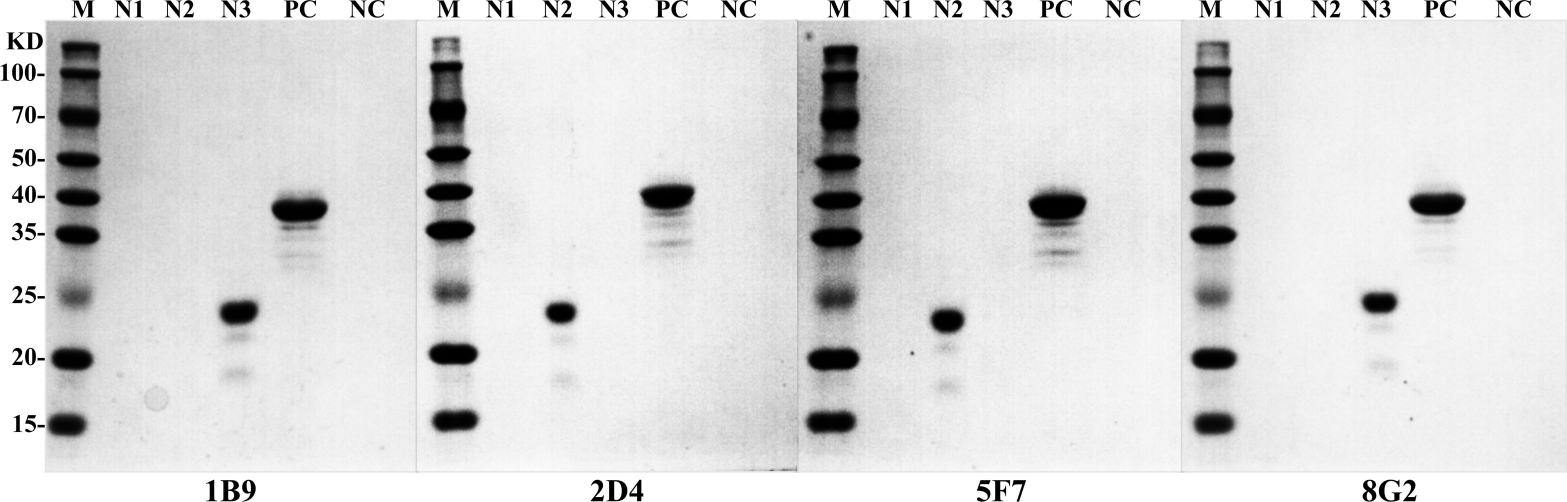
Reactivity of monoclonal antibodies with H171R protein fragments. Western blot results demonstrating the affinity of monoclonal antibodies 1B9, 8G2, 2D4, and 5F7 for the N1, N2, and N3 fragments. In all panels, PC represents the full-length H171R protein as a positive control, and NC represents the TrxA tag protein as a negative control.

**Fig 7:**
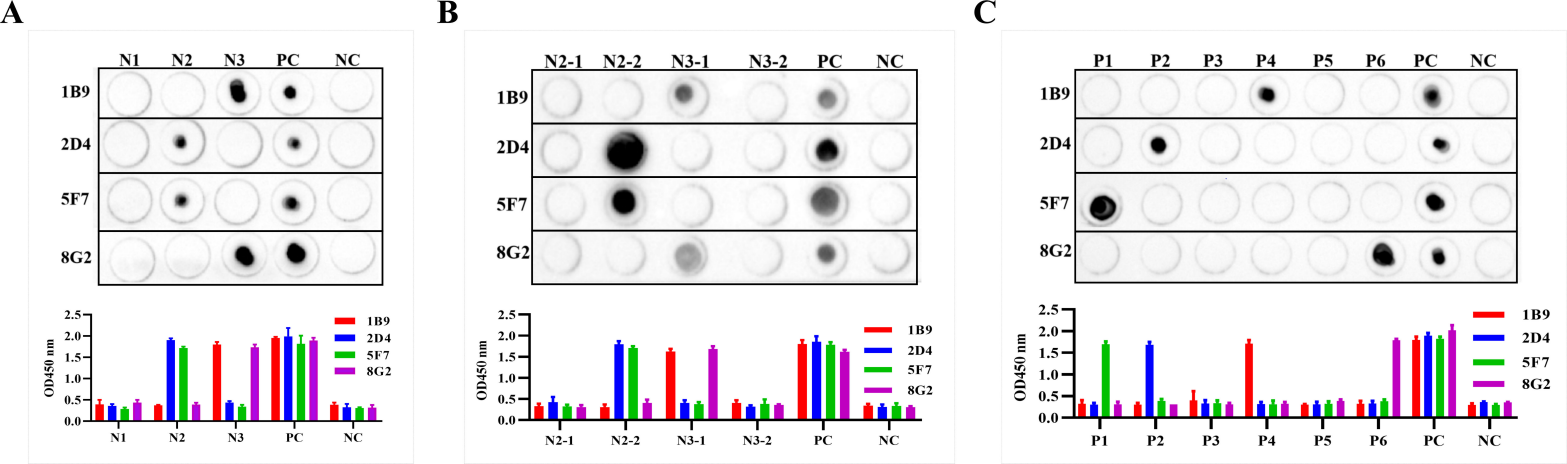
Identification of linear B-cell epitope regions on the H171R protein. (**A**) Reactivity of monoclonal antibodies with N1, N2, and N3 fragments. (**B**) Reactivity of monoclonal antibodies with N2-1, N2-2, N3-1, and N3-2 fragments. (**C**) Reactivity of monoclonal antibodies with P1, P2, P3, P4, P5, and P6 peptides, determining the minimal linear epitopes. In all panels, PC represents the full-length H171R protein as a positive control, and NC represents the TrxA tag protein as a negative control.

To locate the epitope regions recognized by the four monoclonal antibodies, the full-length amino acid sequence of the H171R protein was divided into three larger fragments (N1–N3). Overlapping primers were designed, and these linear peptide fragments were cloned into the pET-32a(+) vector for expression and purification. WB, Dot-ELISA, and ELISA results demonstrated that monoclonal antibodies 1B9 and 8G2 exhibited strong affinity for the N3 fragment, while 2D4 and 5F7 displayed strong binding to the N2 fragment (Fig. 6 and 7A).

Based on the initial results, the N2 and N3 regions were further divided into shorter fragments, N2-1 and N2-2, and N3-1 and N3-2, respectively. Repeated Dot-ELISA and indirect ELISA analyses revealed that mAbs 2D4 and 5F7 primarily recognized the N2-2 fragment, while mAbs 1B9 and 8G2 mainly recognized the N3-1 fragment (Fig. 7B).

Based on these findings, the N2-2 and N3-1 regions were further divided, and six shorter overlapping peptides, P1–P6, were designed with five amino acid overlaps between them. Through Dot-ELISA, ELISA, and immunoprecipitation experiments, the minimal linear epitopes were finally determined: mAb 1B9 reacted with P4, mAb 2D4 reacted with P2, mAb 5F7 reacted with P1, and mAb 8G2 reacted with P6 (Fig. 7C).

**Fig 8:**
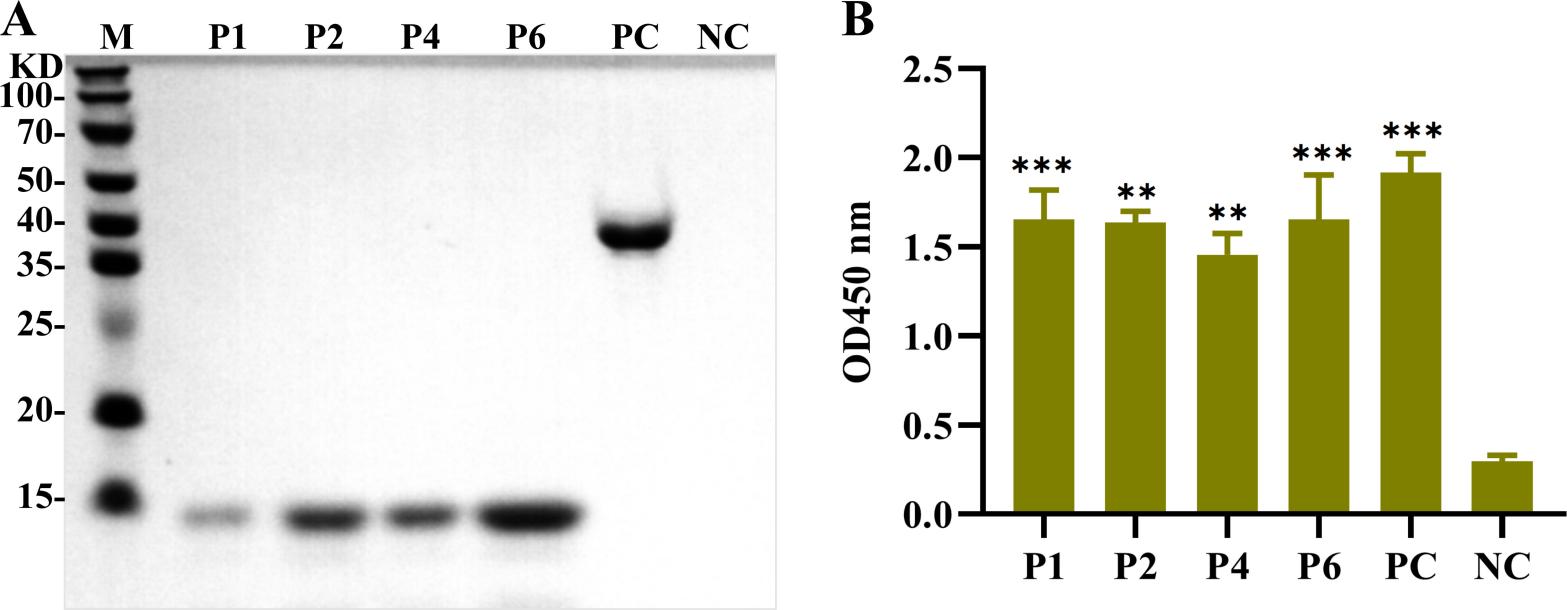
Reactivity of linear epitopes with ASFV-positive pig sera. (**A**) Western blotting results showing the specific reaction of ASFV-positive sera with the four linear epitopes. (**B**) Indirect ELISA results confirming the immunoreactivity of the epitopes with ASFV-positive sera. In both panels, PC represents the full-length H171R protein as a positive control, and NC represents the TrxA tag protein as a negative control.

We further assessed the reactivity of the four linear epitopes and monoclonal antibodies with sera from ASFV-naturally infected pigs. Western blotting and indirect ELISA results demonstrated that the ASFV-positive sera could specifically react with the four linear epitopes while exhibiting no cross-reactivity with the TaxA tag protein (NC) (Fig. 8A and B). These results confirmed the strong immunogenicity of the identified epitopes.

### Identification of critical amino acid residues in the epitopes

To identify the critical amino acid residues within the epitopes, a series of peptide fragments carrying single amino acid substitutions were constructed, and their reactivity with the corresponding monoclonal antibodies was analyzed (Table 2).

**TABLE 2:** Alanine scanning mutagenesis of peptides P1, P2, P4, and P6

P1	Sequence	P2	Sequence	P4	Sequence	P6	Sequence
H84A	**A**PLLPYQQSSDEQP	S93A	**A**DEQPMMPYQQPPG	P111A	**A**YEQIYHKKHASQQ	L129A	**A**NDYYQHILALGDED
P85A	H**A**LLPYQQSSDEQP	D94A	S**A**EQPMMPYQQPPG	Y112A	P**A**EQIYHKKHASQQ	N130A	L**A**DYYQHILALGDED
L86A	HP**A**LPYQQSSDEQP	E95A	SD**A**QPMMPYQQPPG	E113A	PY**A**QIYHKKHASQQ	D131A	LN**A**YYQHILALGDED
L87A	HPL**A**PYQQSSDEQP	Q96A	SDE**A**PMMPYQQPPG	Q114A	PYE**A**IYHKKHASQQ	Y132A	LND**A**YQHILALGDED
P88A	HPLL**A**YQQSSDEQP	P97A	SDEQ**A**MMPYQQPPG	I115A	PYEQ**A**YHKKHASQQ	Y133A	LNDY**A**QHILALGDED
Y89A	HPLLP**A**QQSSDEQP	M98A	SDEQP**A**MPYQQPPG	Y116A	PYEQI**A**HKKHASQQ	Q134A	LNDYY**A**HILALGDED
Q90A	HPLLPY**A**QSSDEQP	M99A	SDEQPM**A**PYQQPPG	H117A	PYEQIY**A**KKHASQQ	H135A	LNDYYQ**A**ILALGDED
Q91A	HPLLPYQ**A**SSDEQP	P100A	SDEQPMM**A**YQQPPG	K118A	PYEQIYH**A**KHASQQ	I136A	LNDYYQH**A**LALGDED
S92A	HPLLPYQQ**A**SDEQP	Y101A	SDEQPMMP**A**QQPPG	K119A	PYEQIYHK**A**HASQQ	L137A	LNDYYQHI**A**ALGDED
S93A	HPLLPYQQS**A**DEQP	Q102A	SDEQPMMPY**A**QPPG	H120A	PYEQIYHKK**A**ASQQ	A138G	LNDYYQHIL**G**LGDED
D94A	HPLLPYQQSS**A**EQP	Q103A	SDEQPMMPYQ**A**PPG	A121G	PYEQIYHKKH**G**SQQ	L139A	LNDYYQHILA**A**GDED
E95A	HPLLPYQQSSD**A**QP	P104A	SDEQPMMPYQQ**A**PG	S122A	PYEQIYHKKHA**A**QQ	G140A	LNDYYQHILAL**A**DED
Q96A	HPLLPYQQSSDE**A**P	P105A	SDEQPMMPYQQP**A**G	Q123A	PYEQIYHKKHAS**A**Q	D141A	LNDYYQHILALG**A**ED
P97A	HPLLPYQQSSDEQ**A**	G106A	SDEQPMMPYQQPP**A**	Q124A	PYEQIYHKKHASQ**A**	E142A	LNDYYQHILALGD**A**D
						D143A	LNDYYQHILALGDE**A**

**Fig 9:**
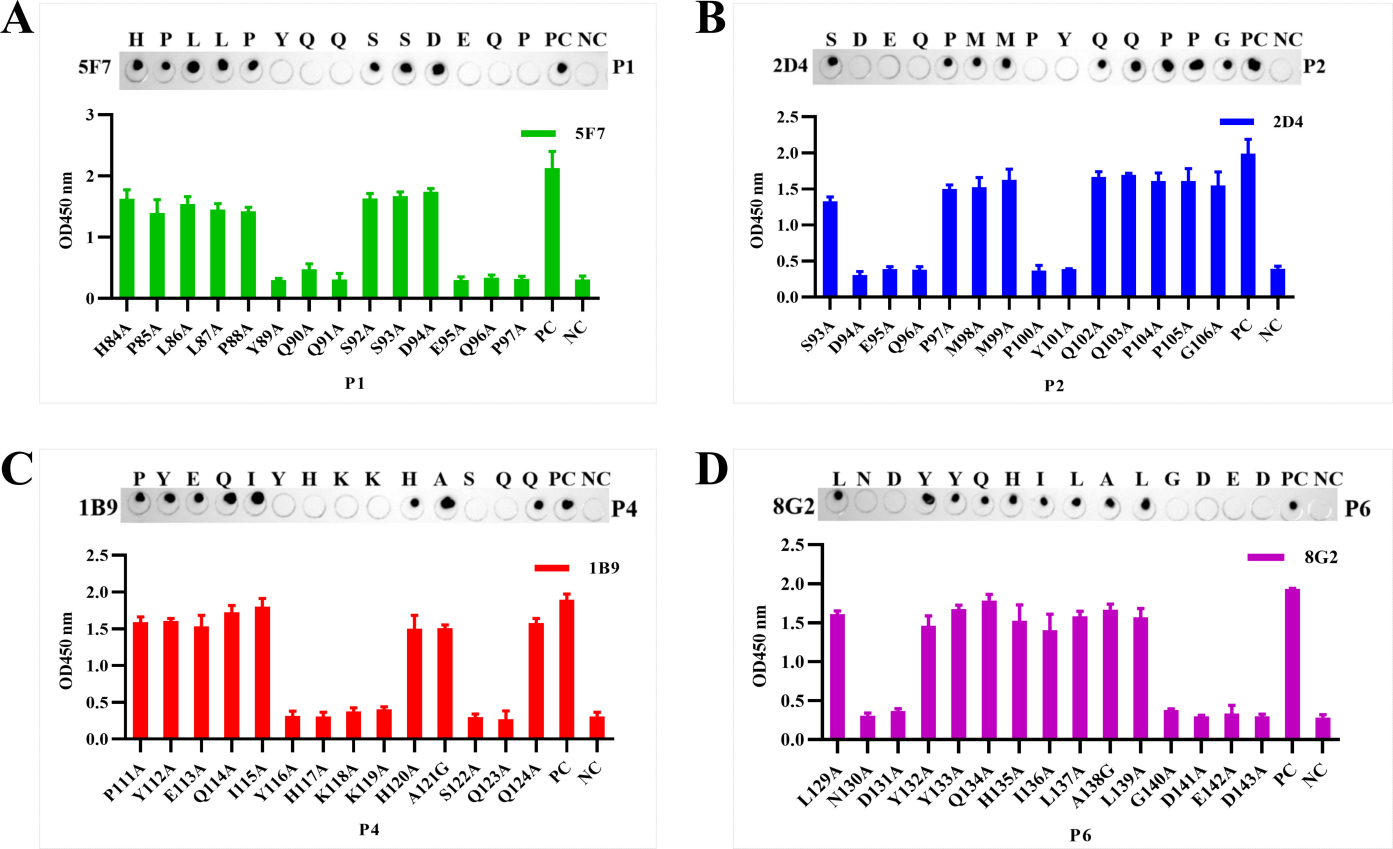
Identification of critical amino acid residues in the epitopes. (**A–D**) Reactivity of monoclonal antibodies 5F7, 2D4, 1B9, and 8G2 with peptide fragments carrying single amino acid substitutions in P1, P2, P4, and P6, respectively.

The results showed that when 89Y, 90Q, 91Q, 95E, 96Q, and 97P were substituted in P1 (^84^HPLLPYQQSSDEQP^97^), the binding ability of mAb 5F7 was significantly reduced. Similarly, when 94D, 95E, 96Q, 100P, and 101Y were substituted in P2 (^93^SDEQPMMPYQQPPG^106^), the affinity of mAb 2D4 was significantly decreased (Fig. 9A and B).

Furthermore, mAb 1B9 was sensitive to mutations at 116Y, 117H, 118K, 119K, 122S, and 123Q in P4 (^111^PYEQIYHKKHASQQ^124^), while mAb 8G2 showed significantly reduced reactivity when 130N, 131D, 140G, 141D, 142E, and 143D were mutated in P6 (^129^LNDYYQHILALGDED^143^) (Fig. 9C and D).

### Distribution of the epitopes on the three-dimensional structure of the H171R protein

**Fig 10:**
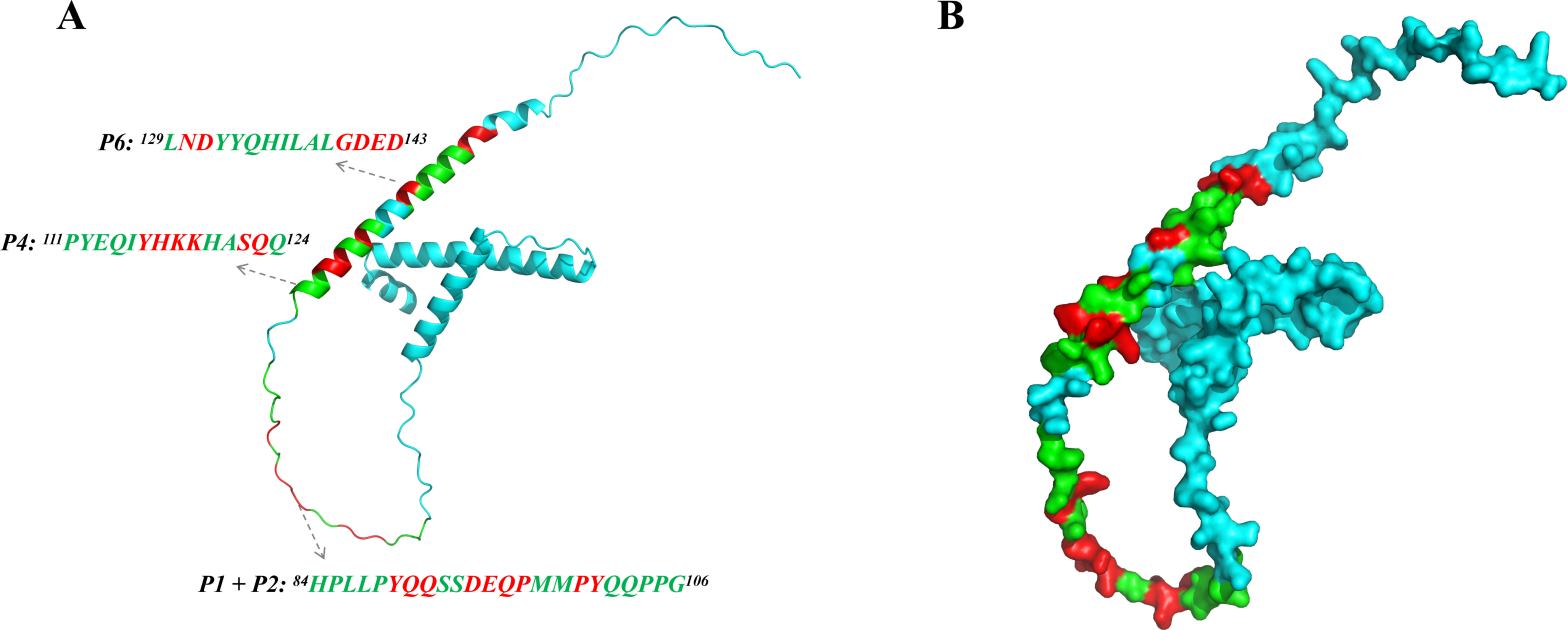
Distribution of the epitopes on the three-dimensional structure of the H171R protein. (**A**) Homology model of the H171R protein constructed using AlphaFold2 software. (**B**) Surface representation of the H171R protein showing the locations of the four identified linear epitopes (^84^HPLLPYQQSSDEQP^97^, ^93^SDEQPMMPYQQPPG^106^, ^111^PYEQIYHKKHASQQ^124^, and ^129^LNDYYQHILALGDED^143^) with critical amino acid residues exposed on the surface.

Using the AlphaFold2 software, we successfully constructed a homology model of the three-dimensional structure of the H171R protein. The four identified linear epitopes ^84^HPLLPYQQSSDEQP^97^, ^93^SDEQPMMPYQQPPG^106^, ^111^PYEQIYHKKHASQQ^124^, and ^129^LNDYYQHILALGDED^143^ were distributed in different regions on the protein surface, with most of the critical amino acid residues exposed on the surface, facilitating their interaction with antibody molecules (Fig. 10A and B). This structural analysis provided important insights for further investigation of the functions of the H171R protein and its interactions with receptors/ligands.

### Conservation analysis of H171R protein epitopes

**Fig 11:**
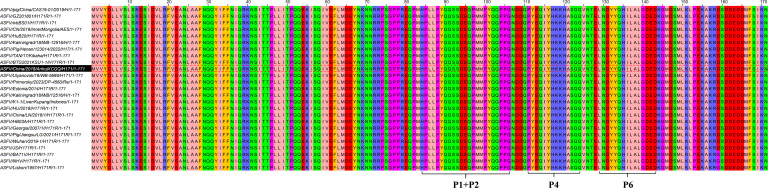
Conservation analysis of the H171R protein epitopes. Multiple sequence alignment of the four epitope regions (P1, P2, P4, and P6) from the selected virus strain with the corresponding sequences from 24 different ASFV isolates, demonstrating complete conservation across the analyzed isolates.

To assess the conservation of the identified epitopes, the amino acid sequences of the four epitope regions in the H171R protein of the selected virus strain were aligned with the corresponding sequences from 24 different ASFV isolates. The alignment results demonstrated that the four epitope regions were completely conserved, with no mutations observed across the analyzed isolates (Fig. 11).

## DISCUSSION

The proteins of H171R are structural proteins involved in the stabilization of virus particles (5). Due to its surface-exposed nature, H171R represents a promising target for developing diagnostic tools and subunit vaccines against ASFV. However, a comprehensive understanding of its antigenic epitopes is crucial for exploiting its potential applications.

In this study, we successfully identified and characterized four minimal linear B-cell epitopes on the H171R protein through a systematic approach combining bioinformatics analysis and experimental techniques. The recombinant H171R protein was expressed and purified, and specific monoclonal antibodies were generated through immunization and hybridoma technology. Epitope mapping using overlapping peptide fragments and alanine scanning mutagenesis revealed the following linear epitopes: ^84^HPLLPYQQSSDEQP^97^, ^93^SDEQPMMPYQQPPG^106^, ^111^PYEQIYHKKHASQQ^124^, and ^129^LNDYYQHILALGDED^143^.

The identification of these linear epitopes has several implications. First, the strong reactivity of the identified epitopes with ASFV-positive swine sera highlights their potential as diagnostic targets for developing serological assays to detect ASFV infection (18). The epitope-specific monoclonal antibodies generated in this study can serve as valuable tools for developing sensitive and specific diagnostic tests, contributing to improved surveillance and control measures for ASF (19).

To comprehensively elucidate the characteristics of the epitopes, we further determined the key amino acid residues in each epitope. The results of tandem mutation and alanine scanning analysis consistently indicated that 116Y, 117H, 118K, 119K, 122S, and 123Q are critical for the 1B9 epitope; 94D, 95E, 96Q, 100P, and 101Y are the key residues of the 2D4 epitope; 89Y, 90Q, 91Q, 95E, 96Q, and 97P have the greatest impact on the 5F7 epitope; and 130N, 131D, 140G, 141D, 142E, and 143D are the main antigenic determinants of the 8G2 epitope. It should be noted that most of these key residues we identified are distributed in the central positions of the epitopes, which is consistent with the theoretical expectations of antigenic determinants of linear epitopes.

The elucidation of critical amino acid residues within each epitope provides valuable insights into the antigenic determinants of the H171R protein (20). This information can guide the design and optimization of epitope-based subunit vaccines or peptide-based immunotherapeutics against ASFV (21). By incorporating these immunodominant epitopes or their optimized variants, the development of more effective and targeted vaccine candidates becomes feasible (22).

Furthermore, the structural modeling and visualization of the H171R protein, with the identified epitopes mapped onto the three-dimensional structure, offer a valuable resource for future investigations into the protein’s functions and interactions. The spatial distribution and accessibility of the epitope regions may provide clues about the potential binding sites for receptors or ligands involved in viral entry, assembly, or immune evasion mechanisms (23).

While the findings of this study provide valuable insights into the linear B-cell epitopes of the H171R protein, there are certain limitations that should be acknowledged. Firstly, the epitope mapping approach employed focused solely on linear epitopes, which may not fully represent the antigenic landscape of the protein. Conformational epitopes, formed by discontinuous amino acid sequences brought into spatial proximity by protein folding, may also contribute significantly to the antigenicity and functional interactions of the H171R protein (24). Future investigations should aim to characterize these conformational epitopes using complementary techniques, such as phage display or co-crystallization with antibody fragments (25).

In summary, we comprehensively identified and characterized four major linear B-cell epitopes of the ASFV H171R protein and their key amino acid residues in this study. These findings not only help elucidate the function of the H171R protein but also lay the theoretical foundation for the future development of novel diagnostic reagents and subunit vaccines for ASFV.

### Conclusion

In conclusion, this study systematically identified and characterized the major linear B-cell epitopes and their key residues of the ASFV key structural protein H171R, laying the foundation for further in-depth research on the immunological characteristics and functional mechanisms of this protein. At the same time, the obtained monoclonal antibodies and epitope information also provide important materials for future development of ASFV detection reagents and design of novel subunit vaccines, which have important theoretical significance and application value for controlling the spread of African swine fever epidemics.
